# Supplementary material for: Age-related differences in negative cognitive empathy but similarities in positive affective empathy
Source: Brain Struct Funct. 2021 May 26;226(6):1823–40. doi: 10.1007/s00429-021-02291-y (PMC8203543; doi:10.1007/s00429-021-02291-y)
Supplement: Supplementary file 1 — Supplementary file1 (DOCX 692 KB) [file 429_2021_2291_MOESM1_ESM.docx]

# Supplementary Materials

# *Ratings of Task Stimuli*

Original ratings of the images used in the MET by Mazza and colleagues (2015): Negative (mean ± SD valence: 2.49 ± 0.18; mean arousal:5.48 ± 0.11; e.g., battered woman, attack, grieving, riot; IAPS number: 3181; 2141; 2095, 2457, 2276, 2301, 2458, 2710, 2750, 2130, 2399, 6311, 2120, 8241, 9332), positive, (mean ± SD valence: 7.72 ± 0.21; mean arousal: 5.03 ± 0.27; e.g., attractive infants, family, romantic images; IAP number: 2071, 2306, 2035, 2304, 7325, 2000, 2010, 2030, 8120, 8350, 8300, 2510, 2511) and neutral pictures (mean ± SD valence: 5.61 ± 0.21; mean arousal: 3.17 ± 1.92; e.g., neutral faces of woman, man and child; IAPS number: 2240, 2270, 2280, 2250, 2385, 2440, 2441, 2200, 2215, 2630, 2305, 2372, 2383, 2394, 2221, 2499, 2500, 2512, 2520, 2513, 2516).

# *Reaction Times: Normality Tests and Distributions*

**Supplementary Table 1.** Results of Kolmogorov-Smirnov and Shapiro-Wilk normality tests on response times by experimental/valence condition and age group

| **Tests of Normality** | | | | | | | |
| --- | --- | --- | --- | --- | --- | --- | --- |
| Age Group | Experimental/Valence  Condition | *Kolmogorov-Smirnov^a^* | | | *Shapiro-Wilk* | | |
|  |  | *Statistic* | *df* | *Sig.* | *Statistic* | *df* | *Sig.* |
| Younger Participants | *Cognitive*  *Empathy* |  |  |  |  |  |  |
|  | NEGATIVE | .120 | 26 | .200^*^ | .952 | 26 | .260 |
|  | POSITIVE | .198 | 26 | .010 | .870 | 26 | .003 |
|  | NEUTRAL | .147 | 26 | .154 | .952 | 26 | .258 |
|  | *Affective Empathy* |  |  |  |  |  |  |
|  | NEGATIVE | .181 | 26 | .028 | .748 | 26 | .000 |
|  | POSITIVE | .203 | 26 | .007 | .825 | 26 | .000 |
|  | NEUTRAL | .089 | 26 | .200^*^ | .944 | 26 | .165 |
|  | *Age Perception (Control)* |  |  |  |  |  |  |
|  | NEGATIVE | .111 | 26 | .200^*^ | .981 | 26 | .885 |
|  | POSITIVE | .143 | 26 | .180 | .887 | 26 | .008 |
|  | NEUTRAL | .163 | 26 | .074 | .860 | 26 | .002 |
| Older Participants | *Cognitive*  *Empathy* |  |  |  |  |  |  |
|  | NEGATIVE | .173 | 24 | .062 | .957 | 25 | .387 |
|  | POSITIVE | .179 | 24 | .044 | .900 | 25 | .022 |
|  | NEUTRAL | .107 | 25 | .200^*^ | .967 | 25 | .561 |
|  | *Affective Empathy* |  |  |  |  |  |  |
|  | NEGATIVE | .123 | 25 | .200^*^ | .975 | 25 | .775 |
|  | POSITIVE | .148 | 25 | .166 | .920 | 25 | .052 |
|  | NEUTRAL | .072 | 25 | .200^*^ | .964 | 25 | .491 |
|  | *Age Perception (Control)* |  |  |  |  |  |  |
|  | NEGATIVE | .152 | 25 | .142 | .915 | 25 | .039 |
|  | POSITIVE | .141 | 25 | .200^*^ | .910 | 25 | .030 |
|  | NEUTRAL | .120 | 25 | .200^*^ | .917 | 25 | .043 |
| *Notes*. * Indicates lower bound of true significance; ^a^ Lilliefors Significance Correction | | | | | | | |

***Reaction Times Distribution***

Younger participants

| 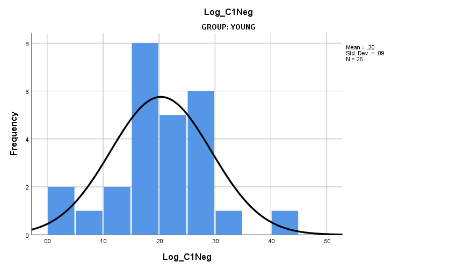 | 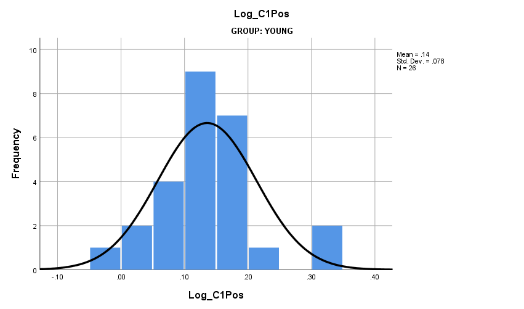 | 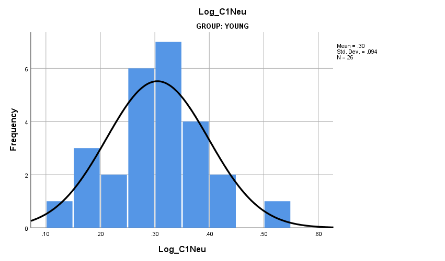 |
| --- | --- | --- |
| 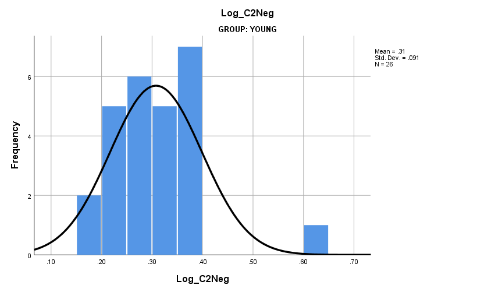 | 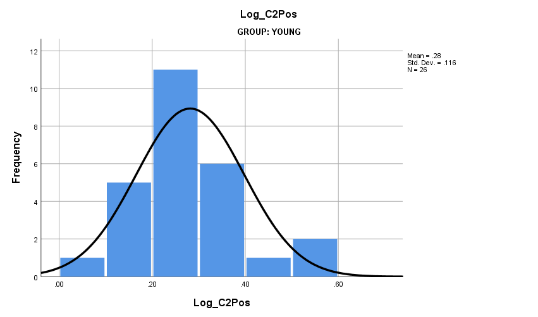 | 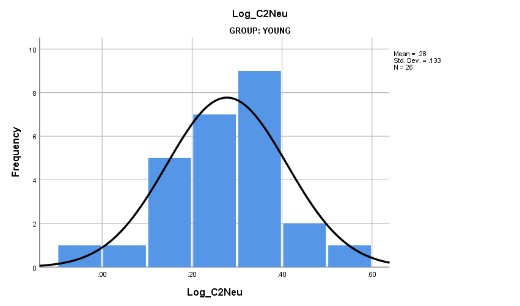 |
| 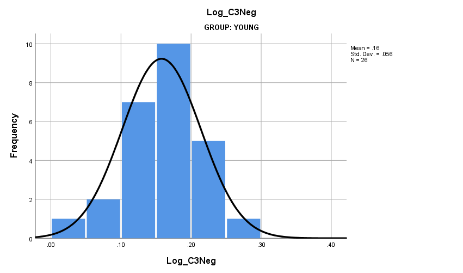 | 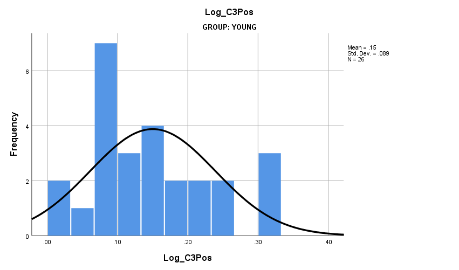 | 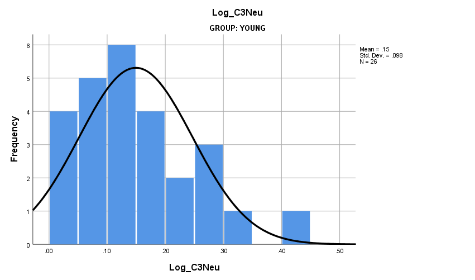 |

Older Participants

| 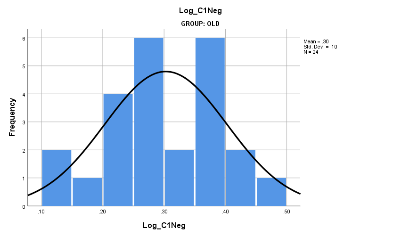 | 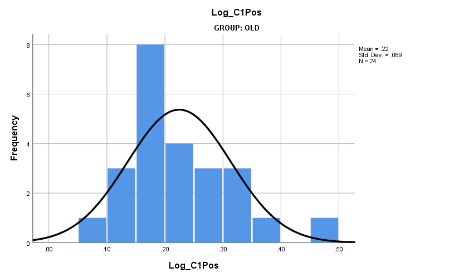 | 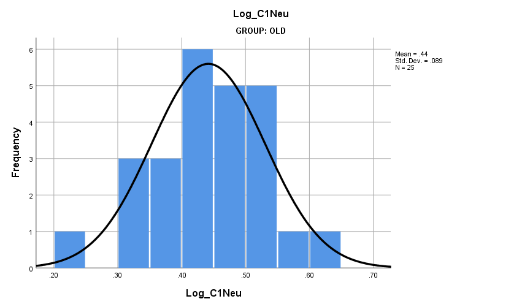 |
| --- | --- | --- |
| 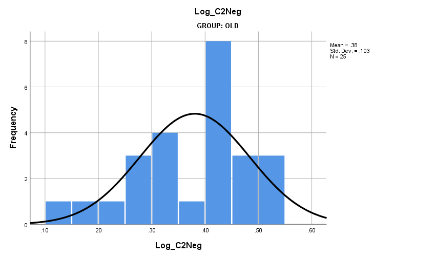 | 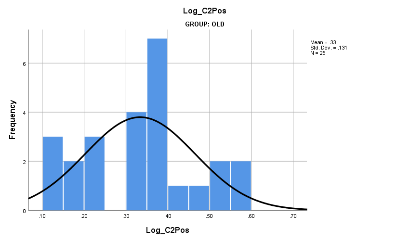 | 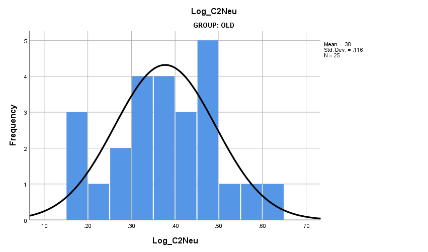 |
| 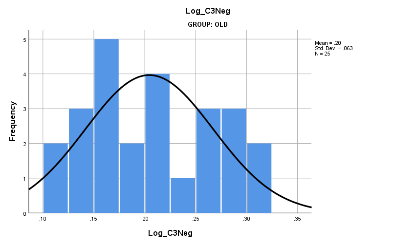 | 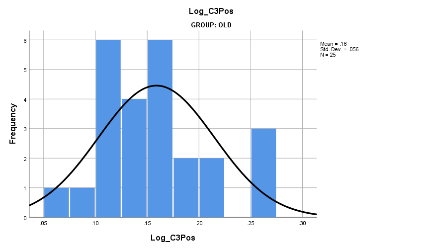 | 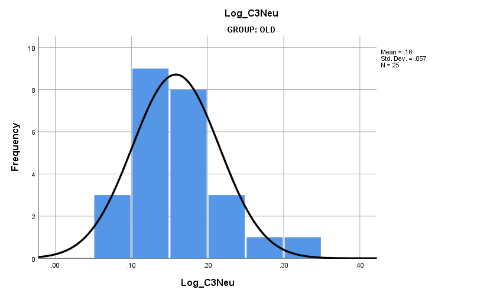 |

***Supplementary Figure 1.*** Distributions of log transformed response times, by experimental/valence condition and age groups. *Notes.* C1 = cognitive empathy; C2 = affective empathy; C3 = age perception; Neg = negative stimuli; Pos = positive stimuli; Neu = neutral stimuli

# *Accuracy: Behavioral Results*

For the cognitive empathy and age perception conditions, accuracy was calculated as the percentage of accurate responses (i.e., correct facial emotion recognition and correct age estimation, respectively). For the affective empathy condition, scores ranged between 7-21, with higher scores indicating higher affective responding (i.e., an accuracy measure per se was not assessed in this condition).

For *cognitive empathy*, the main effect of valence was significant (*F*(2,98) = 48.04, *p* < 0.001, $\eta_{p}^{2}$ = 0.49), indicating that both age groups were more accurate for positive than negative (*t*(50) = 4.67, *p* < 0.001, *d* = 1.32) or neutral (*t*(50) = 8.21, *p* < 0.001, *d* = 2.32) emotions. The main effect of age group was also significant (*F*(2,98) = 3.02, *p* = 0.053, $\eta_{p}^{2}$ = 0.58), in that older participants were less accurate than younger participants.

For *affective empathy*, the main effect of valence was significant (*F*(2,98) = 36.67, *p* < 0.001, $\eta_{p}^{2}$ = 0.42), suggesting that both age groups showed higher affective responding to positive (*t*(50) = 9.72, *p* < 0.001, *d* = 2.74) and negative (*t*(50) = 6.55, *p* < 0.001, *d* = 1.85) than neutral emotions.

For *age perception*, the main effect of valence (*F*(2,98) = 67.99, *p* < 0.001, $\eta_{p}^{2}$ = 0.58) and the interaction between valence and age group (*F*(2,98) = 3.10, *p* = 0.049, $\eta_{p}^{2}$ = 0.06) were significant. Follow-up simple contrasts revealed that older participants were more accurate for positive than negative (*t*(24) = 6.56, *p* < 0.001, *d* = 2.67) or neutral (*t*(24) = 7.89, *p* < 0.001, *d* = 3.22) emotions. Similarly, for younger participants, accuracy for positive (*t*(25) = 4.90, *p* < 0.001, *d* = 1.96) and neutral (*t*(25) = 5.80, *p* < 0.001, *d* = 2.32) emotions was higher than for negative stimuli, but accuracy to positive and neutral emotions did not significantly differ (*t*(25) = 1.17, *p* = 0.25, *d* = 0.46) in this age group.


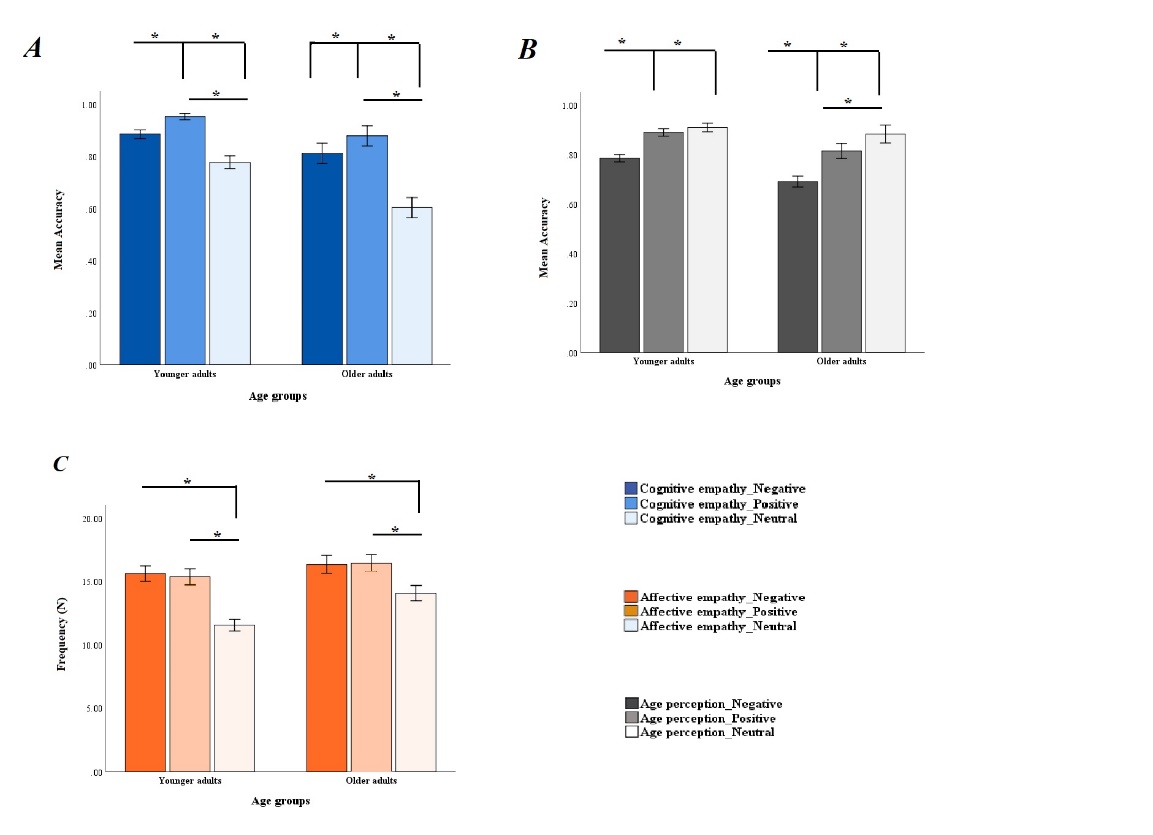


***Supplementary Figure 2*.** Panel A. Mean accuracy for cognitive empathy to positive, negative, and neutral stimuli in younger and older participants. Panel B. Mean accuracy for age perception for positive, negative, and neutral stimuli in younger and older participants. Panel C. Frequency of responses for the weak, average, strong response option during affective empathy to positive, negative, and neutral stimuli in younger and older participants.

# *Brain-Behavior Results on Background Measures*

We included background measures such as the DASS, Stroop, RMET, as well as affective components of the IRI to explore factors involved in age-related differences in affective empathy for positive emotions, on the levels of brain and behavior. We conducted several analyses (for each background measures separately) considering age group and emotional valence in the models.

*Empathic concern and personal distress* – Additional analyses using the four subscales of the IRI (Davis, 1983) revealed a brain pattern that was positively correlated with the empathic concern subscale among younger adults and the personal distress subscale among older adults during affective empathy to positive emotions. In particular, younger adults with high empathic concern scores and older adults with high personal distress scores activated a network that included the bilateral superior temporal gyrus, left medial frontal gyrus, bilateral precentral gyrus, bilateral middle frontal gyrus, anterior cingulate, cuneus, and precuneus.

*DASS* – Our control analyses with the three DASS subscales revealed a brain pattern which was positively correlated with stress, anxiety, and depression scores among younger adults during affective empathy to negative emotions. This network included the anterior and posterior cingulate cortex, cuneus and precuneus, superior temporal gyrus, and superior frontal gyrus. In particular, younger adults with higher total DASS scores activated these areas during negative affective empathy. This network was furthermore correlated with anxiety and depression scores in older adults during positive affective empathy. That is, older adults with higher anxiety and depression scores activated these areas more during positive empathy.

*RMET* – Our analyses with the RMET scores revealed a network which was positively correlated with RMET scores during affective empathy to positive stimuli among older adults. This network included medial and superior frontal gyrus, bilateral inferior parietal lobe, right insula and inferior frontal gyrus areas. Older adults with higher RMET scores engaged this network a larger extend than younger adults during positive affective empathy.

*Stroop* – our analyses with Stroop effect score and MET task revealed a pattern which was positively correlated with the stroop effect among older adults in all conditions of the affective empathy. This network included cuneus, left post central, and bilateral insula regions.

# *Age-Related Differences in Structural Tracts*

We conducted statistical analyses to compare younger and older adults on white matter tract FA values. Results from these analyses suggested no age-related differences in posterior cingulum FA values in either the left (*t*(49) = 0.02, *p* = 0.98, *d* = 0.005 ) or the right (*t*(49) = 0.38, *p* = 0.70, *d* = 0.10) hemisphere. The age groups also did not differ in their left anterior cingulum FA values (*t*(49) = 1.87, *p* = 0.07, *d* = 0.53), but younger participants showed significantly greater FA values in the right anterior cingulum than older participants (*t*(49) = 2.60, *p* = 0.012, *d* = 0.74).

# *Structure-Function Analyses*


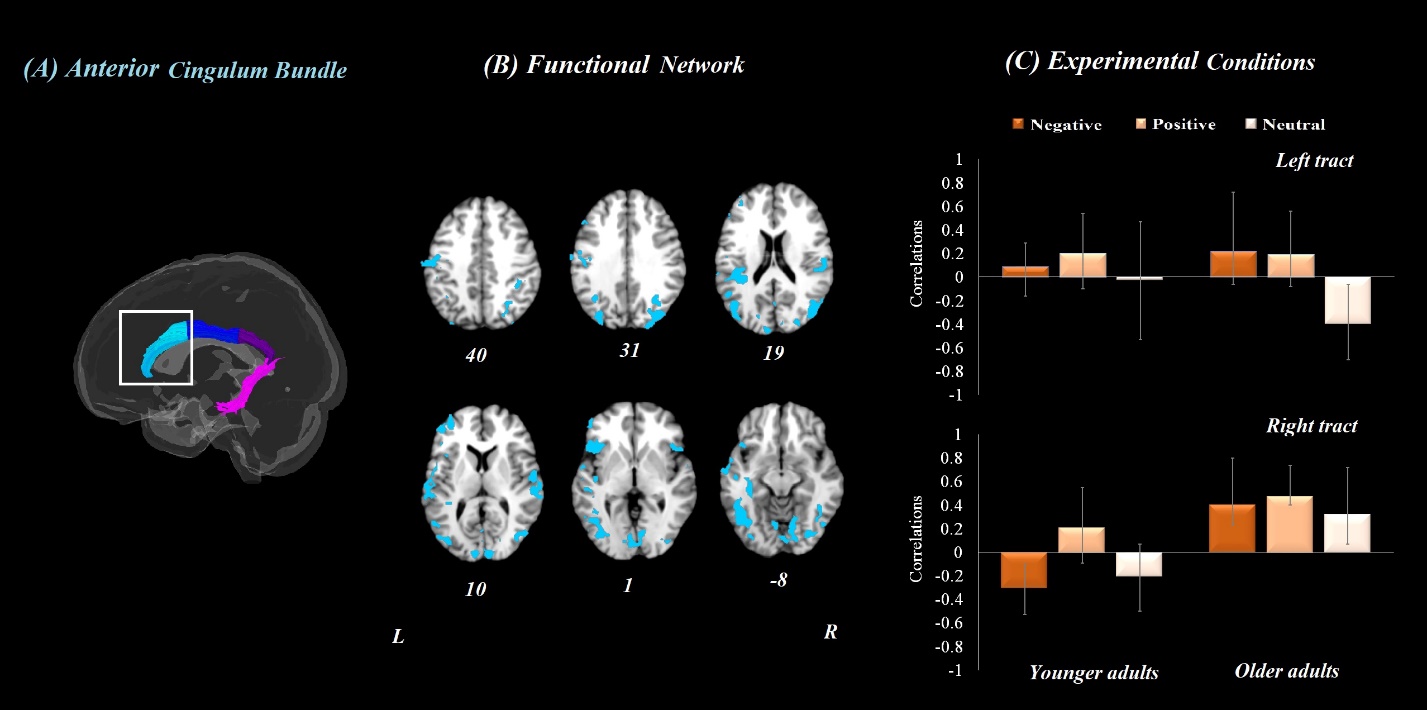


***Supplementary Figure 3.*** Results from the structure-function whole-brain analysis for anterior cingulum fractional anisotropy (FA) values in both age groups for the affective empathy condition. Panel A depicts the anterior cingulum bundle tract. Panel B refers to the brain activation pattern which was correlated with the anterior cingulum bundle FA values the during affective empathy. Panel C refers to the correlation between anterior cingulum bundle FA values and brain patterns for the three valence conditions in both younger and older adults. Error bars represent confidence intervals at 95%. For all reported regions a bootstrap ratio of ≥ 2.5 and cluster size of ≥ 50 voxels was applied. L = left hemisphere, R = right hemisphere.

# *Movement Parameters for the Neuroimaging Data*


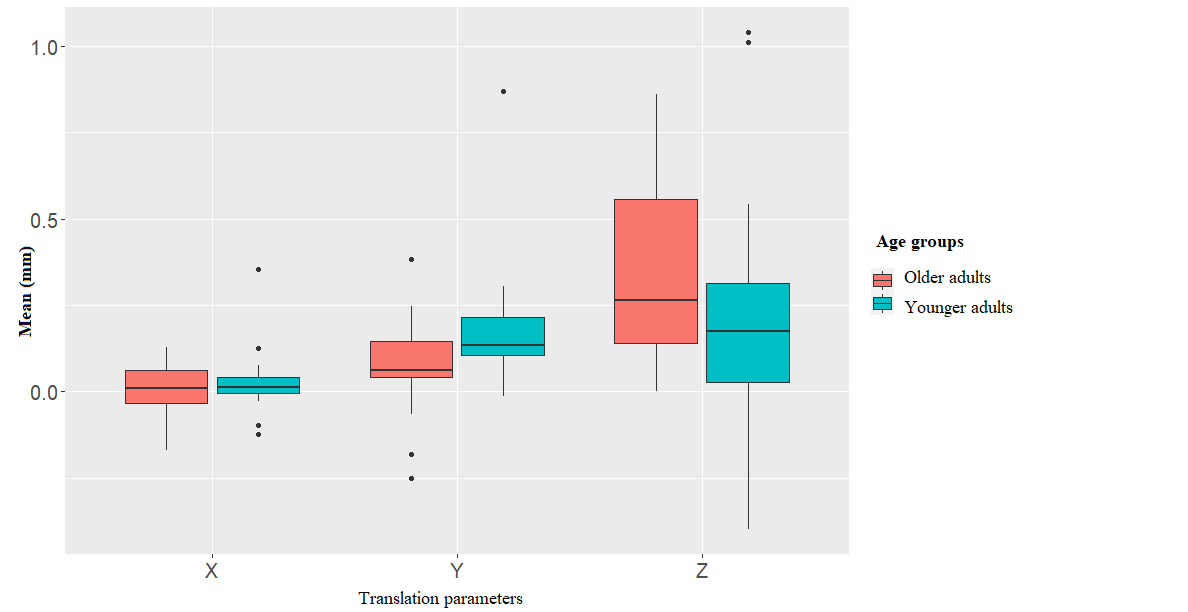

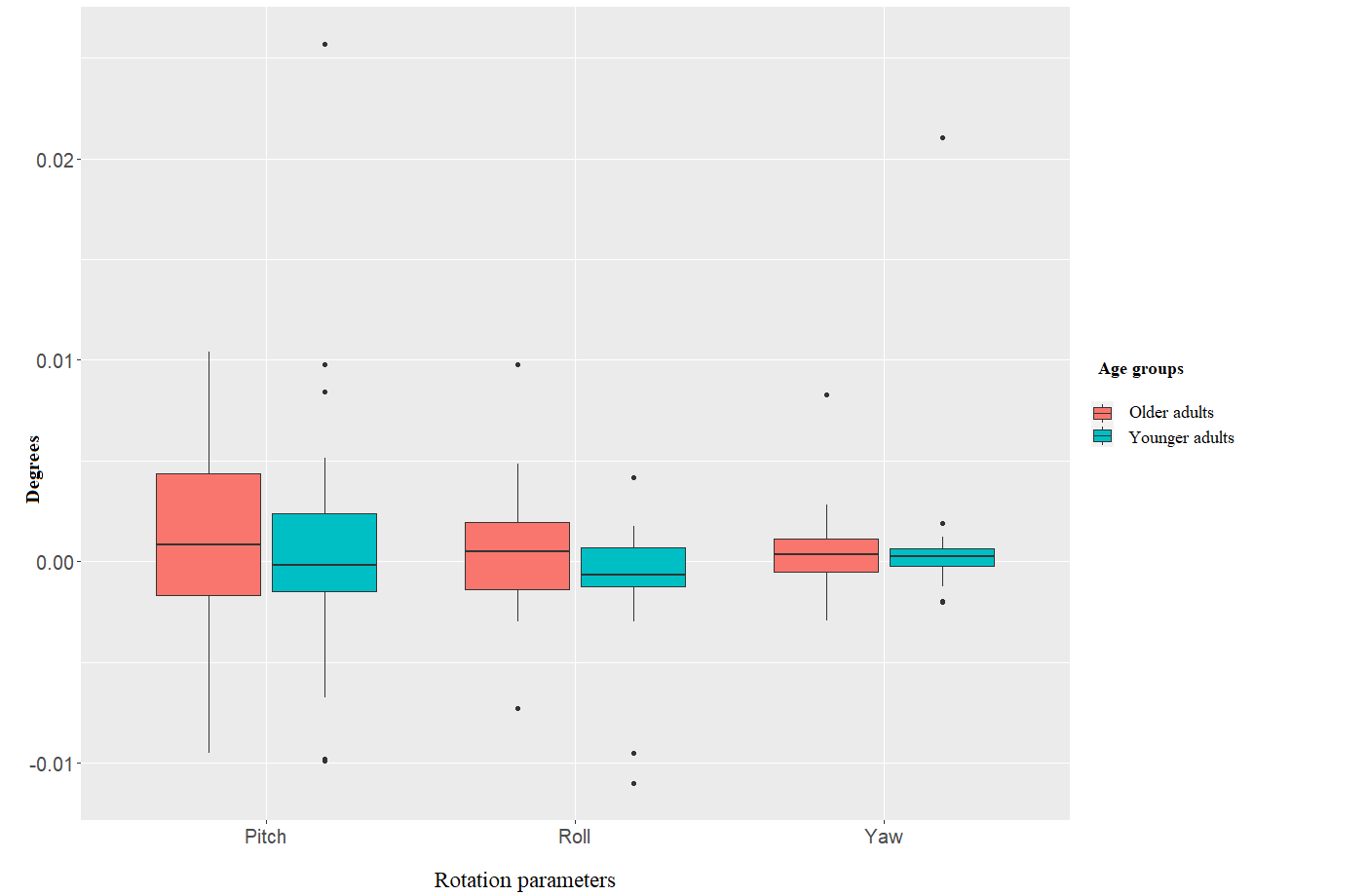


***Supplementary Figure 5.*** Movement parameters across all participants for x, y, z, pitch, roll, and yaw parameters

# *Scatter Plots for Structure-Function Analyses*

Anterior cingulum bundle:


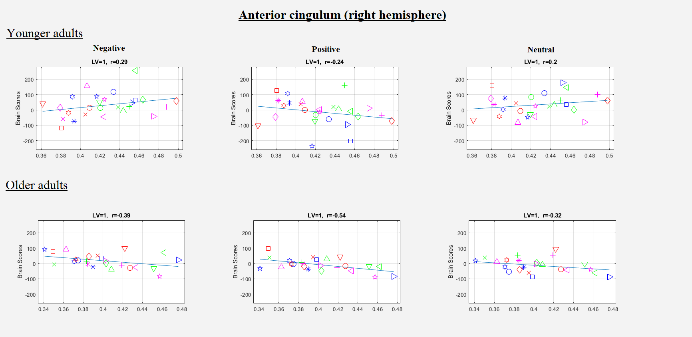


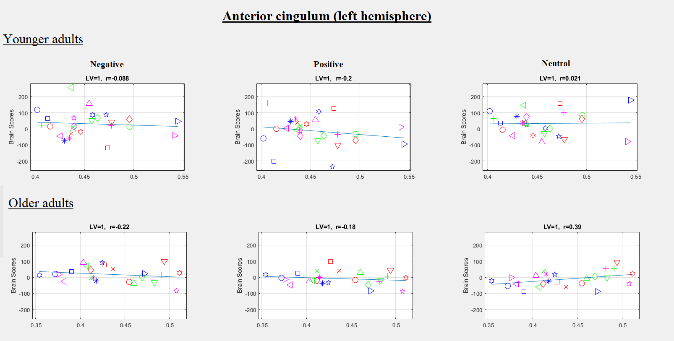


Posterior cingulum bundle


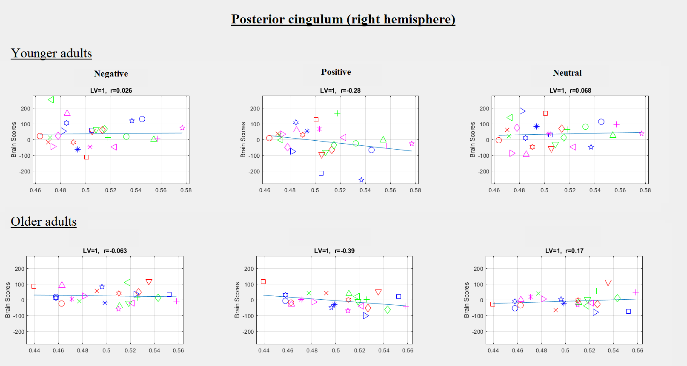


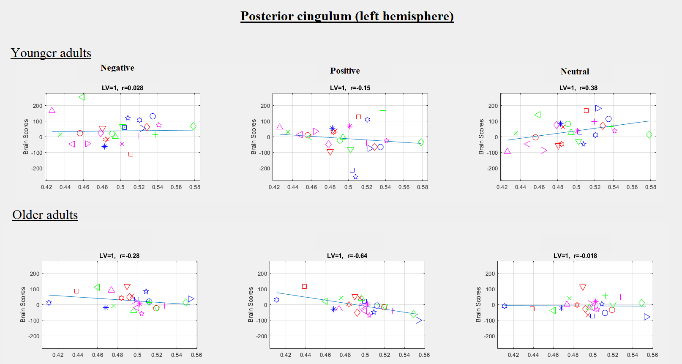


***Supplementary Figure 6.*** Scatter plots for structure-function analyses with anterior and posterior cingulum bundles for each emotional valence during affective empathy condition among older and younger adults
